# Nanoliposomal Nitroglycerin Exerts Potent Anti-Inflammatory Effects

**DOI:** 10.1038/srep16258

**Published:** 2015-11-20

**Authors:** Soroush Ardekani, Harry A. Scott, Sharad Gupta, Shane Eum, Xiao Yang, Alexander R. Brunelle, Sean M. Wilson, Umar Mohideen, Kaustabh Ghosh

**Affiliations:** 1 Department of Bioengineering, University of California, Riverside, CA; 2 Department of Physics and Astronomy, University of California, Riverside, CA; 3 Center for Perinatal Biology, Loma Linda University School of Medicine, Loma Linda, CA

## Abstract

Nitroglycerin (NTG) markedly enhances nitric oxide (NO) bioavailability. However, its ability to mimic the anti-inflammatory properties of NO remains unknown. Here, we examined whether NTG can suppress endothelial cell (EC) activation during inflammation and developed NTG nanoformulation to simultaneously amplify its anti-inflammatory effects and ameliorate adverse effects associated with high-dose NTG administration. Our findings reveal that NTG significantly inhibits human U937 cell adhesion to NO-deficient human microvascular ECs *in vitro* through an increase in endothelial NO and decrease in endothelial ICAM-1 clustering, as determined by NO analyzer, microfluorimetry, and immunofluorescence staining. Nanoliposomal NTG (NTG-NL) was formulated by encapsulating NTG within unilamellar lipid vesicles (DPhPC, POPC, Cholesterol, DHPE-Texas Red at molar ratio of 6:2:2:0.2) that were ~155 nm in diameter and readily uptaken by ECs, as determined by dynamic light scattering and quantitative fluorescence microscopy, respectively. More importantly, NTG-NL produced a 70-fold increase in NTG therapeutic efficacy when compared with free NTG while preventing excessive mitochondrial superoxide production associated with high NTG doses. Thus, these findings, which are the first to reveal the superior therapeutic effects of an NTG nanoformulation, provide the rationale for their detailed investigation for potentially superior vascular normalization therapies.

Loss of endothelium-derived nitric oxide (NO), which prevents leukocyte-endothelial cell (EC) adhesion, is strongly implicated in chronic inflammation associated with debilitating cardiovascular conditions such as pulmonary arterial hypertension (PAH)^1^, atherosclerosis^2^, and diabetes^3^. Administration of nitrates/nitrites, which rapidly produce NO, is thus being explored as anti-inflammatory therapy^4^,^5^. Since organic nitrates exert superior NO-dependent vasodilatory effects when compared with inorganic nitrates/nitrites^6^, they likely also exhibit more potent anti-inflammatory effects.

Of the clinically used organic nitrates, nitroglycerin (NTG) holds particular promise as an anti-inflammatory drug because, in addition to spontaneously producing NO via mitochondrial aldehyde dehydrogenase (ALDH-2)^7^, it also activates endothelial NO synthase (eNOS)^8^,^9^, the key NO-producing enzyme in ECs that is impaired in inflammatory cardiovascular conditions^3^. However, despite its potential anti-inflammatory properties, NTG presents a conundrum as long-term clinical use of current NTG formulations (e.g. transdermal patches, tablets) results in excessive mitochondrial superoxide generation that leads to a loss of NTG sensitivity (tolerance) and endothelial dysfunction (cross-tolerance)^10^,^11^,^12^, thereby limiting its therapeutic efficacy. Thus, new NTG formulations are required to fully leverage the anti-inflammatory potential of NTG.

The field of nanomedicine has enabled the development of nanomaterials (liposomes and polymeric nanoparticles) that can greatly improve drug delivery and therapeutic efficacy by simultaneously increasing drug half-life, lowering effective drug dose (IC_50_), and reducing toxic side-effects^13^,^14^. For instance, our previous work has shown that incorporation of genistein within polymeric nanoparticles improves its anti-inflammatory effect by over two orders of magnitude^15^. Thus, such a nanotherapeutic approach has the ability to amplify the potential anti-inflammatory effects of NTG as well as ameliorate the adverse effects associated with contemporary high-dose NTG administration.

Here, we first demonstrate that NTG-derived NO effectively suppresses endothelial activation during inflammation. Further, we developed a new nanoencapsulation approach for effective NTG delivery that exhibits potent anti-inflammatory effects at a dose 70-fold lower than that of free NTG while preventing excessive mitochondrial superoxide production associated with high NTG doses.

## Results

### NTG Exerts Anti-inflammatory Effects on Activated ECs

**Figure 1:**
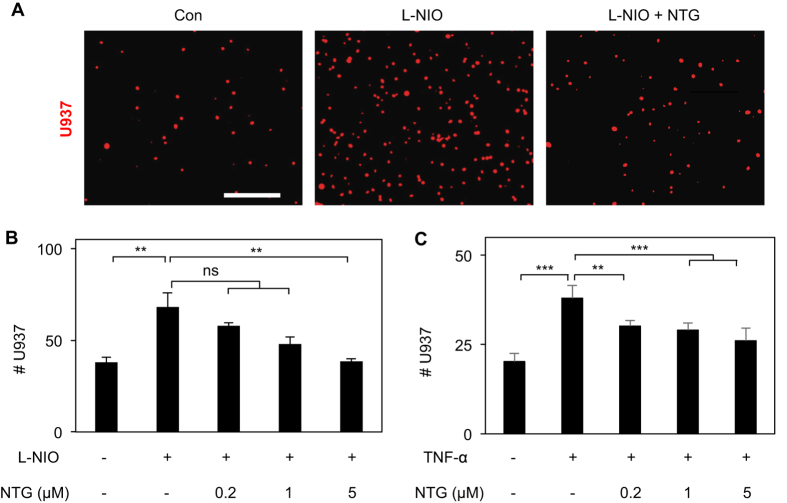
NTG Exerts Anti-inflammatory Effects on Activated ECs. NTG produces dose-dependent inhibition of U937 monocytic cell adhesion to L-NIO-treated (5 mM; overnight) ECs, with significant inhibition observed at 5 μM NTG dose and not lower (0.2 μM and 1 μM doses), as shown in (**A**) the fluorescence images and (**B**) quantified in the bar graph (n = 10 fields of view). **p < 0.01; ns, no significance. (**C**) Similar anti-inflammatory effect of NTG was observed on TNF-α-treated ECs (n = 10 fields of view). **p < 0.01; ***p < 0.001. Data are expressed as mean ± SEM. Scale bar: 200 μm.

Since NTG enhances endothelial NO bioavailability through both spontaneous biotransformation and eNOS activation^7^,^8^, we asked whether NTG could mimic the anti-inflammatory property of NO. To address this question, EC monolayers were treated with L-NIO (5 mM), a selective eNOS inhibitor^8^, or TNF-α (10 ng/ml), which downregulates eNOS mRNA^16^, to impair endogenous endothelial NO production and thereby enhance monocyte-EC adhesion both *in vitro* and in pathological conditions *in vivo*^1^,^3^. Images of fluorescently-labeled adherent U937 cells and their quantification revealed that addition of NTG to L-NIO-treated ECs produces a dose-dependent inhibition of U937 cell adhesion to ECs (Fig. 1A,B), with the inhibition being significant (p < 0.01) at 5 μM NTG dose where U937 cell adhesion was comparable with that on untreated ECs. Notably, NTG exerted a similar dose-dependent anti-inflammatory effect on cells activated with TNF-α (Fig. 1C).

### NTG Enhances Endothelial NO Production

**Figure 2:**
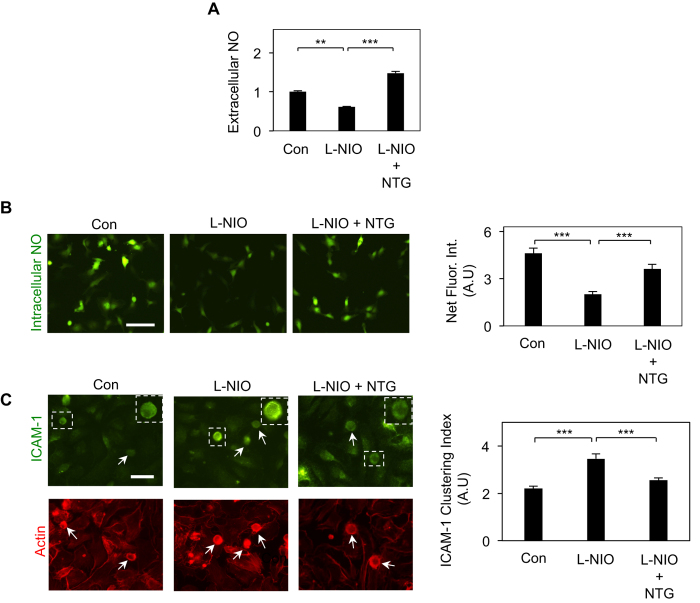
NTG Enhances Endothelial NO Production. (**A**) Addition of NTG (5 μM) to L-NIO-treated ECs prevents the loss of endothelial NO production by L-NIO, as determined by NOA measurement of extracellular NO (n = 4 replicates per condition). Data normalized with respected to untreated control condition (con). **p < 0.01; ***p < 0.001. (**B**) Fluorescent images of ECs labeled with NO-sensitive dye (DAF-FM diacetate) and subsequent image analysis (bar graph; n = at least 30 cells) confirms that NTG-mediated increase in NO results from greater endothelial NO synthesis. *Scale bar:* 25 μm. ***p < 0.001. (**C**) Staining of U937 cell-EC co-cultures with anti-ICAM-1 and phalloidin (to label actin) and subsequent image analysis (bar graph; n ≥ 30 cells), as described in Materials and Methods, indicates that the anti-inflammatory effect of NTG (5 μM) correlates strongly with its ability to prevent ICAM-1 clustering induced by NO deficiency (L-NIO treatment). ***p < 0.001. *Scale bar:* 25 μm. Data are expressed as mean ± SEM.

The potent and hitherto-unknown anti-inflammatory effect of NTG expectedly resulted from an increase in endothelial NO production, as confirmed by two independent approaches. Measurement of released (extracellular) NO by nitric oxide analyzer (NOA) revealed that addition of NTG to L-NIO-treated ECs prevented the loss of NO by L-NIO (Fig. 2A). These findings were independently confirmed by measurement of intracellular NO using a fluorescent NO-sensitive DAF-FM diacetate dye where quantification of fluorescence intensities revealed that NTG causes a significant (79%; p < 0.001) recovery of NO (Fig. 2B).

NO is known to suppress leukocyte-EC adhesion by inhibiting the clustering and/or expression of endothelial cell adhesion molecules (CAMs)^17^,^18^. Our flow cytometry measurement revealed that neither inhibition of NO (using L-NIO) nor its enhancement (using NTG) altered ICAM-1 expression (Supplementary Fig. S1). However, quantitative analysis of fluorescent images of U937 cell-EC co-cultures labeled with anti-ICAM-1 antibody and phalloidin (which stains actin cytoskeleton) revealed that NTG prevents the significant increase (1.6-fold; p < 0.001) in ICAM-1 clustering seen on NO-deficient (L-NIO-treated) ECs (Fig. 2C).

### Synthesis and Physicochemical Characterization of Nanoliposomal NTG (NTG-NL)

**Figure 3:**
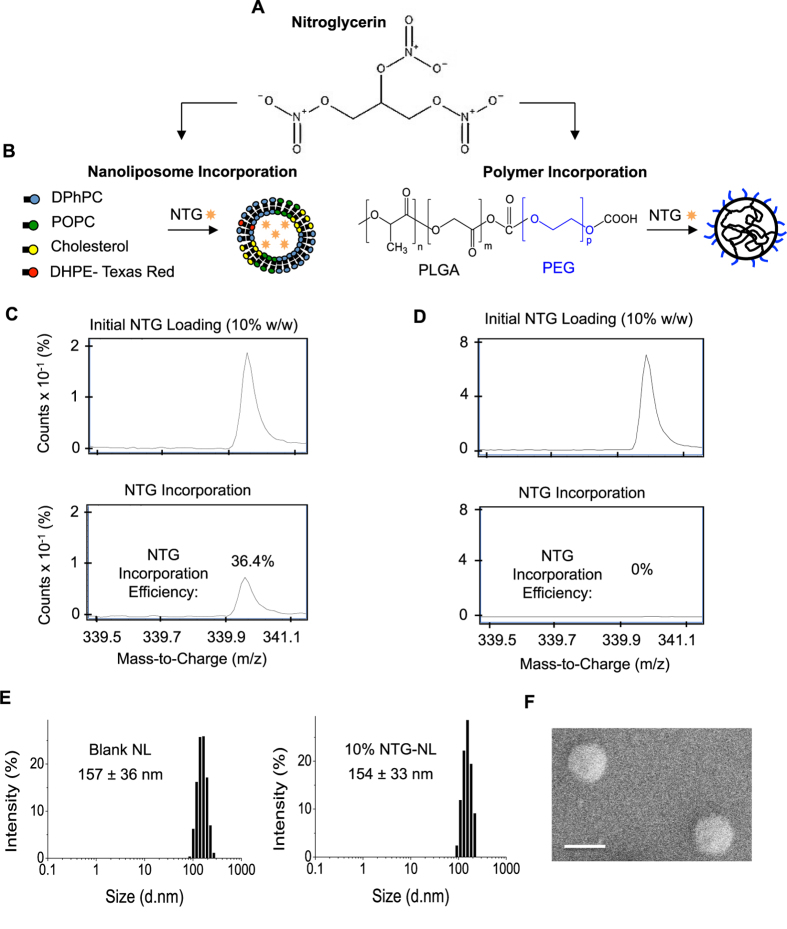
Synthesis and Physicochemical Characterization of Nanoliposomal NTG (NTG-NL). (**A**) Schematic of NTG molecule depicting the hydrophobic (-CH_2_-CH_2_-) and hydrophilic (ONOO^−^) groups. (**B**) For incorporation within nanoliposomes (NLs), NTG was mixed with four lipids viz. DPhPC, POPC, Cholesterol, and DHPE-Texas Red, which self-assemble to form nanoliposomes in an aqueous solution. For incorporation within polymeric NPs, NTG was mixed with an amphiphilic PLGA-b-PEG block copolymer that spontaneously self-assembles in aqueous solution to form NPs with a hydrophobic PLGA core and hydrophilic PEG surface. ESI-Mass Spec. measurements reveal that at 10% w/w initial NTG loading, (**C**) NLs exhibit successful incorporation (~37% incorporation efficiency), while (**D**) polymeric NPs failed to incorporate NTG. (**E**) Dynamic Light Scattering (DLS) analysis reveals that both blank and NTG-loaded NLs exhibit similar diameter (~155 nm). (**F**) Size distribution of NL was independently confirmed using scanning electron microscope (SEM). *Scale bar:* 200 nm. Data are expressed as mean ± Std Dev.

Currently available NTG formulations that aim to treat severe vasoconstriction (angina pectoris) commonly yield a cumulative plasma NTG concentration of ~90 μM (20 mg/L)^19^,^20^,^21^. Such high NTG doses result in excessive mitochondrial superoxide production that, in turn, leads to impaired NTG sensitivity (tolerance) and endothelial dysfunction^10^,^11^,^12^. Thus to successfully leverage the important therapeutic implications of the anti-inflammatory effects of NTG, we employed the principles of nanotechnology to incorporate NTG within nanoparticles (NPs) as they are known to significantly improve drug efficacy^14^,^15^. NPs were made either from an amphiphilic block copolymer poly(D,L-lactide-co-glycolide)-block-poly(ethylene glycol) (PLGA-b-PEG) or from a combination of four lipids (DPhPC, POPC, Cholesterol, and DHPE-Texas Red). The two distinct building blocks were chosen because the NTG molecule contains both hydrophilic and hydrophobic residues (Fig. 3A). Thus, we reasoned that if NTG exhibits predominantly hydrophobic characteristics, it would preferentially incorporate within the hydrophobic PLGA core of the PLGA-b-PEG nanoparticle; if it exhibits greater hydrophilicity, it would incorporate within the hydrophilic core of the lipid nanoparticle (nanoliposomes; NL) (Fig. 3B).

Analysis of electrospray ionization-mass spectroscopy (ESI-MS) peaks revealed differences in NTG uptake into nanoliposomes (NLs) vs polymeric NPs. While NLs demonstrated a dose-dependent increase in NTG loading (Fig. 3C and Supplementary Table S1), polymeric NPs failed to incorporate NTG (Fig. 3D). These findings, which represent the first attempt to encapsulate NTG within a nanocarrier, identify NLs as the preferred vehicle for NTG packaging and delivery. Interestingly, although NTG incorporation within NLs understandably increased with increasing loading, the incorporation efficiency was highest (~37% of initial NTG added) at an intermediate NTG loading of 10% (w/w) (Supplementary Table S1). Based on these findings, 10% (w/w) NTG-NL were chosen as the preferred nanoformulation for subsequent cell functional studies.

The size distribution profile of NLs, obtained using dynamic light scattering (DLS), revealed an average diameter of 157 ± 36 nm and 154 ± 33 nm for blank and NTG-NL, respectively (Fig. 3E), which was independently confirmed by scanning electron microscopy (Fig. 3F).

### Cellular Uptake of Nanoliposomes (NL)

**Figure 4:**
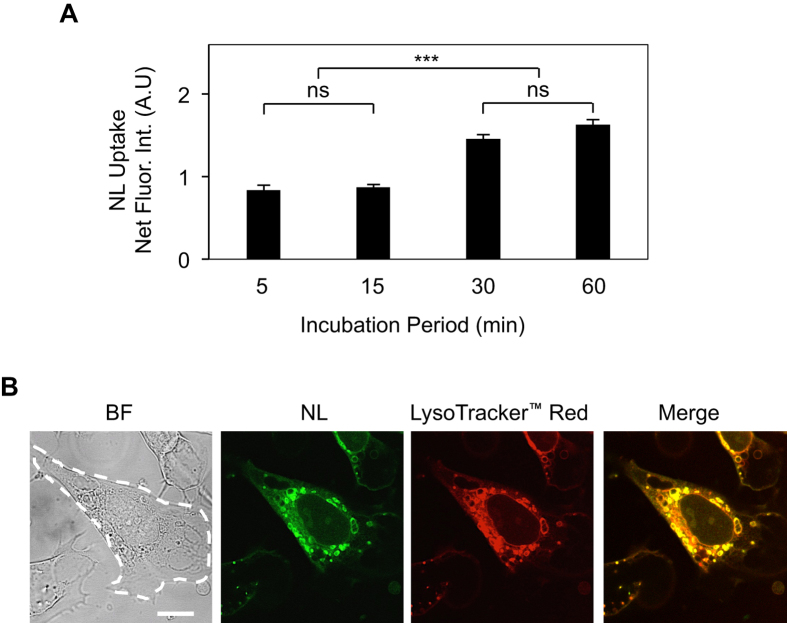
Cellular Uptake of Nanoliposomes. (**A**) Spectrophotometer measurements of internalized fluorescently-labeled nanoliposomes (NL) reveal time-dependent uptake of NLs by cultured ECs (n = 5 replicates per condition). ***p < 0.001; ns, no significance. (**B**) Fluorescent images of internalized NLs indicate strong colocalization with LysoTracker Red^™^-labeled endocytic vesicles (lysosomes and endosomes). *Scale bar:* 10 μm. Data are expressed as mean ± SEM.

Following NL synthesis, we examined their uptake by cultured ECs. Fluorescein-loaded NLs (5 μg/ml) were added to ECs for 5, 15, 30, or 60 min prior to fixation and imaging. Quantification of intracellular fluorescence intensity measurements revealed that nanoliposomal uptake peaked at approximately 30 min, followed by a plateau between 30–60 min (Fig. 4A). These internalized NLs expectedly localized within the acidic endocytic organelles viz. lysosomes and endosomes that line the perinuclear region (Fig. 4B). Notably, although the amount of internalized NLs increased at higher NL doses, the percent internalization was highest (9%) at 5 μg/ml (Supplementary Table S2). Based on these observations of nanoliposomal uptake, all subsequent *in vitro* cell functional studies were performed following 30 min treatment with a 5 μg/ml dose of NL.

### NTG-NL Exerts Superior Anti-inflammatory Effects

**Figure 5:**
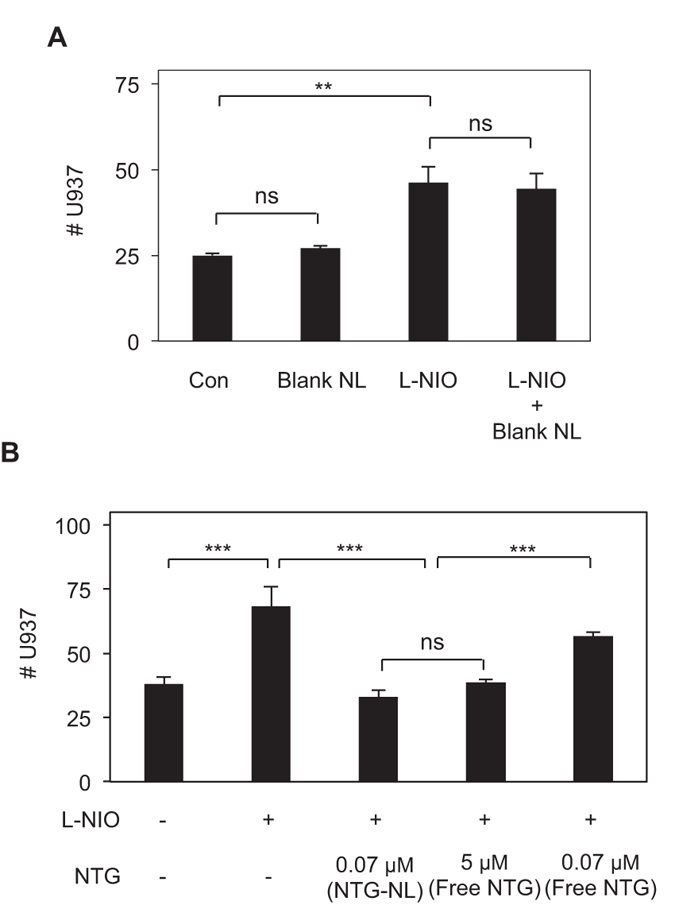
NTG-NL Exerts Superior Anti-inflammatory Effects. (**A**) Blank NLs are inert to ECs as cells treated with blank NLs exhibit neither an increase in U937 cell adhesion (with respect to control) nor a decrease in L-NIO-induced U937 cell adhesion (n = 10 fields of view). **p < 0.01; ns, no significance. (**B**) Addition of 5 μg/ml NTG-NL (≡0.07 μM NTG) to L-NIO-treated ECs significantly inhibits U937 cell-EC adhesion, which is comparable to the inhibition produced by a 70-fold greater dose of free NTG (5 μM). Free NTG dose of 0.07 μM does not produce a significant anti-inflammatory effect. (n = 10 fields of view). ***p < 0.001; ns, no significance. Data are expressed as mean ± SEM.

For NTG-NL to be truly effective as an anti-inflammatory therapy, it is essential that blank NLs exert no inflammatory effects. To confirm this, we analyzed U937 cell adhesion to ECs treated with blank NLs. Quantification of adherent U937 cells revealed that U937 cell adhesion to blank NL-treated ECs is comparable to that seen on untreated ECs (Fig. 5A). Further, treatment of ECs with blank NLs failed to suppress L-NIO-induced increase in U937 cell-EC adhesion (Fig. 5A). These data clearly indicate that blank NLs are totally inert to ECs.

**Figure 6:**
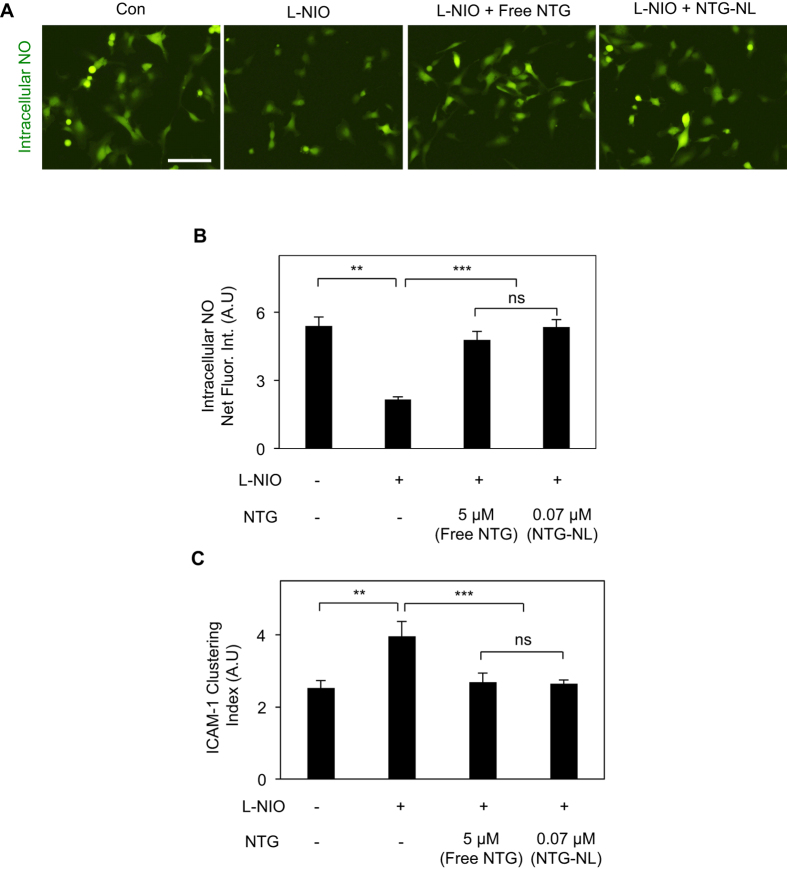
NTG-NL Enhances Endothelial NO Production. (**A**) Immunofluorescent staining of ECs labeled with NO-sensitive dye (DAF-FM diacetate) and (**B**) subsequent image analysis (bar graph; n = at least 30 cells) confirms that, like free NTG, NTG-NL enhances NO production in L-NIO-treated cells. *Scale bar:* 25 μm. **p < 0.01; ***p < 0.001; ns, no significance. (**C**) Immunofluorescent staining of U937 cell-EC co-cultures for ICAM-1 and subsequent fluorescent intensity measurement (bar graph; n = at least 20 cells) reveals that, like free NTG (5 μM), NTG-NL (5 μg/ml) suppresses ICAM-1 clustering induced by NO deficiency (L-NIO treatment). **p < 0.01; ***p < 0.001; ns, no significance. Data are expressed as mean ± SEM.

Nanoliposomes are known to enhance drug efficacy (i.e. reduce effective drug dose) by simultaneously increasing drug half-life and facilitating rapid cellular uptake^13^,^14^,^15^. Thus, we asked whether the internalized NTG-NLs could improve the anti-inflammatory effect of incorporated NTG. Our studies indicate that addition of NTG-NL at 5 μg/ml to L-NIO-treated ECs produced a significant (52%; p < 0.001) prevention of U937 cell adhesion to ECs, with the number of adherent U937s returning to the levels seen on untreated ECs (Fig. 5B). This reduction in U937 cell-EC adhesion by NTG-NL was comparable to the anti-inflammatory effect produced by a 5 μM (0.284 μg total) dose of free NTG. Determination of the total amount of NTG delivered intracellularly through nanoliposomal formulation revealed that NTG-NL produced its potent anti-inflammatory effect at an equivalent NTG dose of 0.07 μM (0.004 μg total), which is 70-fold less than the effective dose of free NTG (5 μM) (refer to Materials and Methods). In other words, NTG-NL was found to be 70-fold more effective than free NTG in suppressing endothelial activation. That this remarkable increase in therapeutic efficacy resulted primarily from nanoformulation of NTG was confirmed by the observation that an equivalent amount (0.07 μM) of free NTG failed to produce a significant anti-inflammatory effect (Fig. 5B). NTG-NLs stored at 4 °C and 37 °C for 24 hr exerted similar anti-inflammatory effects (Supplementary Fig. S2), thereby confirming past findings that nanoliposomes act as stable drug carriers^22^. Moreover, similar to free NTG, the anti-inflammatory effects of NTG-NL correlated strongly with its ability to enhance endothelial NO production (Fig. 6A,B) and suppress ICAM-1 clustering (Fig. 6C).

### NTG-NL Prevents Endothelial Superoxide Formation Associated with High NTG Dose

**Figure 7:**
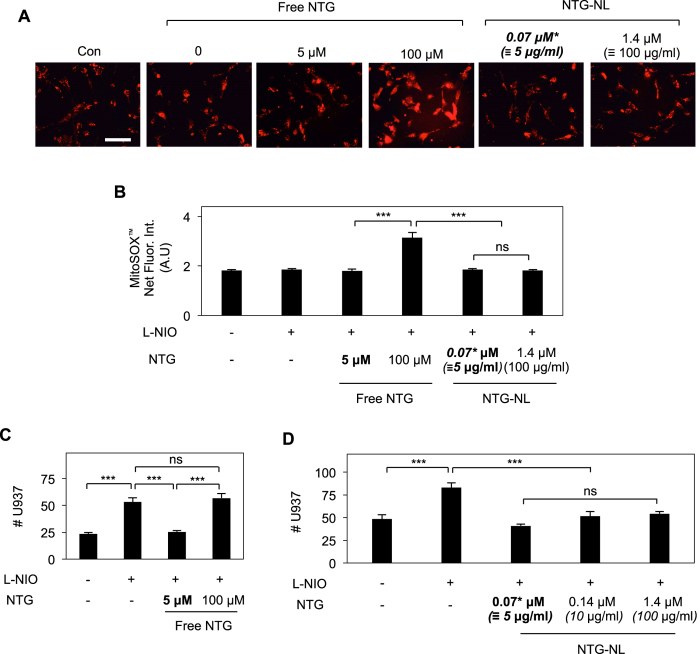
NTG-NL Prevents Endothelial Superoxide Formation Associated with High NTG Dose. (**A**) Representative fluorescent images of MitoSOX^™^-labeled ECs and (**B**) subsequent quantification (bar graph; n ≥ 30 cells) indicate that addition of 20-fold higher dose (100 μM) of free NTG to L-NIO-treated ECs significantly increases mitochondrial superoxide formation while similar increase in NTG-NL dose (0.07 μM to 1.4 μM) produces no effect. *Scale bar:* 100 μm. ***p < 0.001; ns, no significance. (**C**) The high dose (100 μM) of free NTG fails to suppress U937 monocytic cell adhesion to L-NIO-treated ECs (n = 10 fields of view). ***p < 0.001; ns, no significance. (**D**) Unlike free NTG, NTG-NL continues to exert potent anti-inflammatory effects on L-NIO-treated ECs even at a 20-fold higher dose (n = 10 fields of view) with all NTG-NL doses demonstrating similar inhibition of U937 adhesion. Therapeutic dose is indicated ***in bold***. ***p < 0.001; ns, no significance. Data are expressed as mean ± SEM.

High plasma concentrations of NTG that result from the use of conventional NTG formulations (e.g. transdermal patches and tablets) are known to cause significant increase in mitochondrial superoxide formation that, in turn, leads to impaired NTG bioconversion to NO (tolerance) and endothelial dysfunction^10^,^11^,^12^. Consistent with these observations, treatment of ECs with a 100 μM dose of free NTG, which is comparable to the high doses used in NTG therapies, caused a 2-fold (p < 0.001) increase in mitochondrial superoxide formation (Fig. 7A,B), as determined by quantitative analysis of cells labeled with MitoSOX^™^, a fluorescent mitochondrial superoxide-sensitive dye that is widely used to measure mitochondrial reactive oxygen specie (ROS) production in response to high-dose NTG treatment^23^. Notably, as shown in Supplementary Fig. S3, this difference in MitoSOX^™^ fluorescence intensity is a true reflection of the differences in mitochondrial superoxide production and not an artifact resulting from varying cell density or spreading.

Since NTG-NL exhibited potent anti-inflammatory effects at a dose 70-fold lower than that of free NTG, we next asked whether a similar 20-fold increase in NTG-NL dose would ameliorate the adverse effects associated with high-dose NTG therapies. Indeed, we found that NTG-NL did not elicit any increase in mitochondrial superoxide production when used at the 20-fold higher dose of 100 μg/ml (Fig. 7A,B).

Consistent with the view that excessive mitochondrial superoxide production at high NTG doses inhibits ALDH-2 activity and, thus, NTG bioconversion to NO, we observed a drastic loss (~2-fold decrease; p < 0.001) in the anti-inflammatory effects of free NTG at 100 μM (Fig. 7C). In contrast, NTG-NL retained its potent immunosuppressive effects (84% inhibition; p < 0.001) when used at a 20-fold higher dose of 100 μg/ml (Fig. 7D). We further confirmed NTG-NL’s ability to prevent loss of NTG sensitivity at high doses using an *ex vivo* arterial vasorelaxation assay. This assay, which is widely used to evaluate NTG tolerance^10^,^24^, demonstrated that while arteries pretreated with 100 μM free NTG exhibited a significant loss of NTG sensitivity (IC_50_ increasing from 0.2 to 15 μM), those treated with NTG-NL at an equivalent 20-fold higher dose maintained their normal vasodilatory responsiveness (Supplementary Fig. S4).

## Discussion

This study demonstrates a proof-of-principle for a new nanoliposomal NTG formulation that amplifies the newly-identified anti-inflammatory properties of NTG while significantly ameliorating the adverse effects associated with high NTG doses. NTG is one of the most commonly used organic nitrates in the clinic where it is intended to mimic the vasodilatory effects of endothelium-derived NO^19^. When compared with inorganic nitrites/nitrates, NTG produces a significantly greater and rapid yield of NO^6^, which explains its superior vasodilatory properties. Since endothelial NO also exhibits potent anti-inflammatory effects, here we asked whether NTG can suppress leukocyte-EC adhesion.

Our findings reveal that NTG significantly inhibits U937 cell adhesion to NO-deficient ECs. This anti-inflammatory effect of NTG occurred in a dose-dependent manner, with significance achieved at a dose of 5 μM. That a higher NTG dose is required to achieve significant anti-inflammatory effect than is required for vasodilation (IC_50_ = 0.2 μM) may be attributed to the fact that these different therapeutic effects of NTG involve different target cell types with potentially distinct NTG sensitivity. For instance, NTG exerts its anti-inflammatory effect on luminal ECs that undergo activation to promote monocyte adhesion^25^ while its vasodilatory effects are targeted towards abluminal smooth muscle cells that exhibit excessive vasoconstriction^1^. Thus, it is plausible that smooth muscle cells are more sensitive to NTG than ECs, and thereby require lower doses to exhibit NTG-dependent vasodilation. It should, however, be noted the effective anti-inflammatory NTG dose of 5 μM still lies within the dose range (0.09–90 μM) that is widely used for current NTG therapies^19^,^20^,^21^.

Further, consistent with past findings that suppression of monocyte-EC adhesion by NO results from inhibition of endothelial CAM clustering^17^, NTG treatment produced significant inhibition of ICAM-1 clustering on EC surface. ICAM-1 is a key EC surface marker that facilitates leukocyte-EC adhesion by undergoing clustering on EC surface, which results in the formation of an ICAM-1 binding pocket for firm leukocyte docking and adhesion^26^. Past studies have indicated that the ability of NO to inhibit ICAM-1 clustering likely results from suppression of Rho/ROCK-dependent actin cytoskeletal reorganization at the site of monocyte-EC adhesion^27^,^28^.

Although inorganic nitrites have been shown to exert anti-inflammatory effects^4^, to our knowledge, this is the first report indicating that NTG also exhibits similar properties. Coupled with previous findings that (1) NTG activates eNOS^8^,^9^, the major NO-producing enzyme in ECs that is impaired in inflammatory conditions such as PAH^1^ and diabetes^3^, and (2) NTG mimics the anti-coagulating properties of NO to prevent inflammation-associated hypercoagulopathy^29^, the current findings provide further rationale for investigating the use of NTG for anti-inflammatory therapies.

NTG undergoes rapid (within 15–30 min) clearance from bloodstream^30^. To address this issue, current NTG formulations are commonly administered at high doses (up to 90 μM)^19^,^20^,^21^. It is, therefore, likely that similar high NTG doses may be necessary to achieve its potent anti-inflammatory effects. However, this approach poses a limitation in that such high NTG doses lead to excessive mitochondrial superoxide production, which greatly limit NTG’s therapeutic efficacy^10^,^12^. Mitochondrial superoxide, a reactive oxygen species that is formed as a byproduct during NTG bioconversion to NO, inhibits the activity of mitochondrial aldehyde dehydrogenase (ALDH-2)^10^,^12^, the chief enzyme responsible for NTG bioconversion. Thus, excessive amounts of mitochondrial superoxide generated in response to high NTG doses significantly impairs ALDH-2 activity and, as a consequence, NTG bioconversion and sensitivity^12^. Indeed, our studies confirmed that raising the dose of free NTG from 5 μM to 100 μM led to a significant increase in mitochondrial superoxide production that, in turn, correlated with a significant loss in the anti-inflammatory effects of NTG.

To improve the benefit/risk profile of NTG therapy, we leveraged the principles of nanotechnology to develop a new nanoliposomal NTG formulation (NTG-NL) that undergoes rapid intracellular uptake via the conventional clathrin-mediated pathway^31^ and demonstrates a remarkable 70-fold increase in drug efficacy. Such a tremendous increase in drug efficacy is the hallmark of nanocarrier-mediated drug delivery, which is known to enhance drug half-life and promote rapid cellular uptake and bioavailability of encapsulated drugs^14^,^15^. Importantly, the significant enhancement in NTG-NL bioactivity and resultant lowering of the effective therapeutic dose meant that, unlike free NTG, NTG-NL did not elicit an increase in mitochondrial superoxide production even at very high (20-fold greater) dose. This observation is supported by findings that, in contrast to free NTG, NTG-NL treatment at a high dose did not exhibit any loss of anti-inflammatory effect *in vitro* or loss of vasorelaxation response in isolated pulmonary arteries. Taken together, these findings implicate NLs as an effective NTG delivery system that has the potential to amplify the newly-identified anti-inflammatory effects while addressing the adverse effects associated with high NTG doses.

The *in vitro* and *ex vivo* studies performed here are widely used to examine the effects of NTG on the vasculature^12^,^23^. However, to fully establish the translational potential of nanoliposomal NTG, the current proof-of-concept studies need to be validated in appropriate animal models of vascular inflammation. NTG-NL are expected to be biocompatible as the lipids used to synthesize these nanoliposomes are found in cell membranes^32^. This excellent biocompatibility is an important reason why liposomes are widely used as drug carriers in pharmaceutical industry^14^,^32^. Further, the size (~150 nm dia.) of NTG-NL is suitable to achieve a long circulation time^14^,^33^. Thus, the proof-of-concept studies described here set the stage for the development of *site-targeting* NTG nanotherapeutics that can further improve the efficacy of nanoliposomal NTG by simultaneously increasing drug half-life, localizing drug delivery to sites of inflammation, and reducing undesirable off-target effects. As we and others have previously shown^15^,^34^, such site-targeting can be accomplished by tethering unique site-targeting moieties (peptides, aptamers or antibodies)^14^,^15^ on the nanoparticle surface that can guide the nanotherapeutic selectively to desired vascular sites and facilitate local drug delivery and therapeutic effects. Thus, by simultaneously leveraging the newly-identified anti-inflammatory and well-known vasodilatory properties of NTG, such smart nanomedicine approaches may provide a superior therapeutic strategy for effective management of PAH that is characterized by both chronic pulmonary arterial inflammation and vasoconstriction^35^.

## Materials and Methods

### Cell Culture

Human microvascular endothelial cells (HMEC-1) were purchased from the Center for Disease Control (CDC)^36^ and cultured on gelatin-coated tissue culture dishes in growth medium composed of MCDB-131 (VWR International, USA) supplemented with 10% FBS (Fisherbrand, USA), 2 mM L-Glutamine (Invitrogen, USA), 1× antimycotic/antibiotic mixture (Life Technologies, USA), 10 ng/ml huEGF (Millipore, USA) and 1 μg/ml Hydrocortizone (Sigma Aldrich, USA). Human U937 monocytic cells were purchased from ATCC (Manassas, VA, USA) and cultured in suspension in growth medium composed of RPMI 1640 (Fisherbrand, USA) supplemented with 2 mM L-Glutamine (Invitrogen), 10 mM HEPES (Fisherbrand, USA), 10% FBS (Fisherbrand), antimycotic/antibiotic mixture (Life Technologies, USA), 1 mM sodium pyruvate (Life Technologies, USA) and 4.5 mg/ml glucose (Sigma Aldrich, USA).

### Nanoparticle (NP) Formulation

To synthesize NTG-loaded polymeric nanoparticles (NP), block co-polymer PLGA (17 kDa)-PEG-COOH (3.4 kDa) (75:25), Advanced Polymer Materials Inc., QC, Canada) was dissolved in DMSO (Sigma Aldrich, USA) at a final concentration of 1 mg/ml^15^, followed by addition of NTG at 10% w/w. NPs were obtained by dialysis using Spectra/Por 6 dialysis membrane (1 kDa MWCO; VWR, USA) where the NTG-polymer solution was dialyzed against distilled water (5 L) at room temperature, which also removed excess NTG. To synthesize NTG-loaded nanoliposomes (NTG-NL), four lipid molecules viz. 1,2-di-(3,7,11,15-tetramethylhexadecanoyl)-sn-glycero-3-phosphocholine (DPhPC; Avanti Lipids, USA), 1-hexadecanoyl-2-(9Z-octadecenoyl)-sn-glycero-3-phosphocholine (POPC; Avanti Lipids, USA), Cholesterol (Sigma Aldrich, USA), and 1,2-dihexadecanoyl-sn-glycero-3-phosphoethanolamine-triethylammonium salt (Texas Red-DHPE; Invitrogen, USA) were dissolved in chloroform at a molar ratio of 6:2:2:0.2, respectively, and purged with Nitrogen (N_2_) to evaporate the chloroform. The resulting lipid cake was placed under vacuum for at least two hours prior to rehydration in aqueous NTG (5, 10 and 25% w/w of total lipid; Cerilliant, USA) or Fluorescein (1 mM; Sigma Aldrich, USA) solution to obtain a final 1 mg/ml drug- or dye-loaded liposome suspension. To obtain NTG-NLs, these liposome suspensions underwent five freeze-thaw cycles in liquid N_2_ followed by eight extrusion cycles through a 100 nm polycarbonate membrane filter (Avanti Lipids, USA). Unincorporated NTG or fluorescein was discarded by spinning down NTG-NLs for two hours at 60,000 rcf using a refrigerated ultracentrifuge (Beckman Coulter, USA) and decanting the supernatant. The final NTG-NL pellet was suspended at 1 mg/ml in water and stored at 4 °C until use.

### NTG Incorporation Efficiency

To determine NTG incorporation efficiency within polymeric NPs and NTG-NLs, pellets of polymeric NP (1 mg) or NTG-NL (200 μg) were dissolved in 100% methanol and analyzed using electron spray ionization-mass spectroscopy (ESI-MS; Agilent Technologies).

NTG (MW 227.1 Da) was ionized by trifluoroacetic acid (MW: 112.9 Da) and the signature NTG mass/charge spectrum peak was detected at 339.9 mass/charge (m/z; charge z = 1 coulomb). Area under the NTG peak was measured for both the initial and incorporated NTG and their ratio was calculated to determine % NTG incorporation efficiency.

### Nanoliposome (NL) Size and Morphology Characterization

Blank NL and NTG-NL suspensions were prepared at 0.5 mg/ml in distilled water and size distribution measured by dynamic light scattering (DLS) using a Delsa Nano C Particle Analyzer (Beckman Coulter, USA). Microsoft Excel and Origin Pro software were used to acquire and analyze the data. NL morphology was analyzed using scanning electron microscopy (SEM; FEI NNS450) operated in high vacuum mode. For SEM samples, NLs were fixed in 2.5% glutaraldehyde (Electron Microscopy Sciences, USA) for 2 hr at 4 °C. After fixation, 10 ul of NLs were added to Poly-L-Lysine (Sigma-Aldrich, USA)-coated 12 mm glass coverslips, allowed to settle for 5 minutes, and subjected to critical-point drying in liquid CO_2_ (Critical-point-dryer Balzers CPD0202). Samples were then sputter-coated with chromium for 30 sec and analyzed using the SEM instrument described above.

### Nanoliposome (NL) Uptake

To determine the rate of NL uptake by ECs, Texas Red^®^-labeled NLs were diluted in EC culture medium at 5 μg/ml and added to ECs for 5, 15, 30 or 60 min at 37 °C. To quantify the extent of NL uptake at different doses, Texas Red^®^-labeled NLs at 5, 10, 50 or 100 μg/ml were added to EC monolayer for 30 min at 37 °C. After treatment, non-internalized NLs were removed by rinsing ECs twice with PBS, following by culturing ECs in phenol-red free MEM media (Life Technologies, USA) supplemented with 2 mM L-Glutamine and 10% FBS and measurement of fluorescence intensity by Wallac 1420 Victor2 fluorescent microplate plate reader (Perkin Elmer, USA). To determine % NL uptake, fluorescence intensity of NL-treated ECs was divided by the intensity obtained from unrinsed samples. Further, to determine whether NTG-NLs were successfully endocytosed, ECs treated with fluorescein-incorporated NLs were stained with LysoTracker^®^ Deep Red (Invitrogen, USA) to label acidic organelles (lysosomes and endosomes).

### Monocyte-EC Adhesion Assay

To examine the effects of NTG on monocyte-EC adhesion, confluent EC monolayers were either serum-starved (MCDB-131, 2.5% FBS, and 1× antibiotic/antimycotic supplement) overnight and treated with 10 ng/ml TNF-α (eBiosciences, USA) for 4 hr or treated with 5 mM L-NIO (N^5^ - (1- iminoethyl)- L- ornithine; selective eNOS inhibitor; Cayman Chemical, MI, USA) ± varying doses of NTG (0, 0.07, 0.2, 1 or 5 μM; Cambridge Isotope, MA) for overnight in regular medium, followed by addition of fluorescently-labeled U937 monocytic cells at a density of 130,000 cells/cm^2^. To confirm that NTG-NLs can be readily uptaken by ECs and processed to release NO, some EC cultures were also treated with L-NIO ± NTG-NL for 4 hr prior to plating fluorescently-labeled U937 cells. After 30 min of U937 cell -EC interaction at 37 °C, U937 suspension was removed and the EC monolayers gently rinsed twice with PBS (to remove unbound U937s) prior to fixation in 1% paraformaldehyde (PFA; Electron Microscopy Sciences, USA). Fluorescent images (10 per condition) of labeled U937 cells were then acquired using a Nikon Eclipse Ti microscope (Nikon, Japan) fitted with a Nikon Digital Sight DS-Qi1Mc camera and the number of adherent U937s was counted using ImageJ software (NIH). For experiments involving NLs, ECs were incubated with 5 μg/ml of blank or 10% (w/w) NTG-loaded nanoparticles (NTG-NL) for 30 minutes at 37 °C, rinsed with PBS to remove excess NLs, and cultured overnight prior to addition of fluorescently-labeled U937 cells (as described above).

### Determination of NTG-NL’s therapeutic efficacy

The therapeutic efficacy of NTG-NL was determined by taking a ratio of the net *free* NTG dose required to exert significant anti-inflammatory effect with the net NTG dose needed to exert the same effect when delivered within NL. Significant anti-inflammatory effect was achieved at a *free* NTG dose of 5 μM (1.136 μg/ml), which results in a net NTG dose of 0.284 μg per 250 μL of EC culture medium. In comparison, considering a 10% (w/w) initial NTG loading, 36.4% NTG incorporation efficiency within NLs, and a 9% intracellular uptake, NTG-NLs exhibit similar anti-inflammatory effect at a dose of 5 μg/ml (0.182 μg/ml of NTG), which results in a net intracellular NTG dose of 0.004 μg per 250 μL of EC culture medium. The ratio of net *free* NTG dose (0.284 μg) to net NTG dose delivered via *NLs* (0.004 μg) was used to determine the fold-increase in NTG efficacy achieved with NTG-NLs.

### Measurement of EC-derived NO

Two independent methods were used to measure the amount of NO produced by ECs. In the first method, ECs were treated overnight with L-NIO (5 mM) ± NTG (5 μM) or NTG-NL (10 μg/ml), followed by incubation with a NO-sensitive fluorescent dye DAF-FM diacetate (2 μM; Life Technologies, USA) for 20 minutes at 37 °C. Following dye loading, ECs were again treated with L-NIO and NTG during a recovery phase for an additional 1 hr in regular growth medium. EC culture media was then rinsed once with Krebs-Henseleit Buffer (KHB) containing 125 mM NaCl, 4.74 mM KCl, 2.5 mM CaCl_2_, 1.2 mM KH_2_PO_4_ 1.2 mM MgSO_4_, 5 mM NaHCO_3_ and 10 mM Glucose (Sigma Aldrich, USA), replaced with fresh KHB and immediately subjected to live cell fluorescence imaging using Nikon Eclipse Ti microscope. At least 30 cells per condition were analyzed for total cell fluorescence using ImageJ software (NIH). For the second method, a Nitric Oxide Analyzer (NOA; Sievers, USA) was used to measure EC-secreted NO in culture medium. For this measurement, confluent ECs were treated overnight with either L-NIO (5 mM) ± NTG (5 μM) or NTG alone, followed by sequential 1 hr incubations with KHB without and with L-NIO ± NTG at 37 °C. KHB conditioned medium (4 replicates per condition) was then collected and analyzed using NOA.

### ICAM-1 Clustering

ECs were grown to confluence on glass coverslips under normal growth conditions and treated with L-NIO (5 mM) ± [NTG (5 μM) or NTG-NL (5 μg/ml)] for 24 hr. Next, a monocyte adhesion assay was performed (as described earlier) and the U937 cell-EC cocultures fixed, permeabilized with 0.1% Triton X-100, blocked with 2% bovine serum albumin (BSA; Millipore, USA), and sequentially incubated with primary anti-ICAM-1 mouse antibody (Santa Cruz Biotechnology, USA) and secondary FITC-conjugated DyLight 488 anti-mouse IgG (Vector Labs, USA). To visualize actin microfilaments, U937 cell-EC cocultures were incubated with Alexa Fluor 594-Phalloidin (BD Biosciences, USA). Coverslips were mounted onto glass slides and fluorescence images (15 per condition) were taken using a Nikon Eclipse Ti microscope fitted with a Nikon Digital Sight DS-Qi1Mc camera. ICAM-1 clustering index was determined by measuring ICAM-1 fluorescence intensity (from n ≥ 10 images) at the U937-EC adhesion site and normalizing it to the average ‘background’ intensity measured from three neighboring EC cytoplasmic sites.

### ICAM-1 Expression

ECs were plated to confluence under normal growth conditions and treated with L-NIO (5 mM) ± NTG (5 μM) overnight. ECs were then detached and sequentially labeled with primary anti-ICAM-1 antibody (Santa Cruz Biotechnology, USA) for 20 min, followed by FITC-conjugated DyLight 488 anti-mouse IgG (Vector labs. USA). Next, ECs were fixed with 1% PFA, detected by a Cell Lab Quanta SC flow cytometer (Beckman Coulter, CA), and analyzed by FlowJo software (Treestar Inc, CA).

### Detection of Endothelial Mitochondrial Superoxide Production

ECs were plated at sub-confluence on gelatin-coated MatTek dishes under normal culture conditions and subjected to overnight treatment with either L-NIO (3 mM) ± [NTG (5, 25, or 100 μM) or NTG-NL (5, 50 or 100 μg/ml)]. To detect mitochondrial superoxide, ECs were labeled with MitoSOX^™^ (Life Technologies, USA), a mitochondrial superoxide-sensitive fluorescent dye that is widely used to measure mitochondrial ROS production under various conditions, including NTG treatment^23^. ECs were labeled with MitoSOX^™^ Red at a final dose of 5 μM in KHB for 10 min at 37 °C, rinsed three times with KHB to remove excess dye and placed at 37 °C in KHB for an additional 10 min prior to live cell imaging. Fluorescence images (six per condition) were acquired using Nikon Eclipse Ti microscope and total cell fluorescence intensity from at least 20 cells was analyzed using ImageJ software.

### Pulmonary Artery Ring Preparation and Isometric Tension Measurements

All animal procedures were in accordance with the Animal Welfare Act, the Guiding Principles in the Care and Use of Animals approved by the Council of the American Physiological Society, the National Institutes of Health Guide for the Care and Use of Laboratory Animals, and Loma Linda University Institutional Animal Care and Use Committee (IACUC). Pregnant ewes were anesthetized with ketamine (10 mg/kg, IV) and midazolam (5 mg/kg, IV) and anesthesia maintained with inhalation of 1-3% isoflurane in O_2_ throughout surgery as required. Ewes and Fetuses were euthanized with an overdose of Euthasol (pentobarbital sodium, 100 mg/kg) and phenytoin sodium (10 mg/kg; Virbac, Ft. Worth, TX).

To assess arterial vasorelaxation in response to NTG treatment, pulmonary arteries (4^th^–5^th^ order) were harvested from newborn fetal sheep (gestational period between 138–141 days), dissected free of parenchyma and cut into 5 mM long rings (at least eight per condition) in ice-cold HEPES buffer (Sigma Aldrich, USA). Rings were then preincubated in L-NIO (1 mM) ± [NTG (5 or 100 μM) or NTG-NL (5 or 100 μg/ml)] for 4 hr at 37 °C; free NTG at 100 μM has previously been reported to induce NTG tolerance of isolated arterial rings within 4 hr^12^. Following treatment, rings were mounted onto tungsten wires under 0.5 g of resting tension in organ baths containing KHB, gassed with 95% O_2_–5% CO_2_, and maintained at 37 °C. Arterial rings were rinsed once with KHB, allowed to equilibrate for at least 30 minutes, and retensioned prior to addition of 125 mM KCl (Sigma Aldrich, USA) to ensure rings were still functional. Rings were rinsed three times to remove KCl, allowed to relax, and preconstricted with 10 μM Serotonin (Tocris Bioscience, USA) to achieve 100% contraction. Rings were then exposed to increasing concentrations of free NTG (1 nm to 100 μM) and subsequent recordings of concentration-response curves were acquired using a force displacement transducer (AD Instruments, New Zealand) and analyzed using Prism (Graphpad Software Inc).

### Statistics

All data were obtained from multiple replicates (as described in the appropriate sections) and expressed as mean ± standard error of mean (SEM). Statistical significance was determined using analysis of variance (ANOVA; InStat; Graphpad Software Inc.) followed by a Tukey multiple comparison post-hoc analysis. Results demonstrating significance were represented as *p < 0.05, **p < 0.01, or ***p < 0.001.

## Additional Information

**How to cite this article**: Ardekani, S. *et al.* Nanoliposomal Nitroglycerin Exerts Potent Anti-Inflammatory Effects. *Sci. Rep.* **5**, 16258; doi: 10.1038/srep16258 (2015).

## Supplementary Material

Supplementary Information
