# Supplementary material for: Nanoliposomal Nitroglycerin Exerts Potent Anti-Inflammatory Effects
Source: Sci Rep. 2015 Nov 20;5:16258. doi: 10.1038/srep16258 (PMC4653649; doi:10.1038/srep16258)
Supplement: Supplementary Information [file srep16258-s1.pdf]

Nanoliposomal Nitroglycerin Exerts Potent Anti-Inflammatory Effects

Soroush Ardekani, Harry A. Scott, Sharad Gupta, Shane Eum, Xiao Yang, Alexander R. Brunelle, Sean M. Wilson, Umar Mohideen, Kaustabh Ghosh

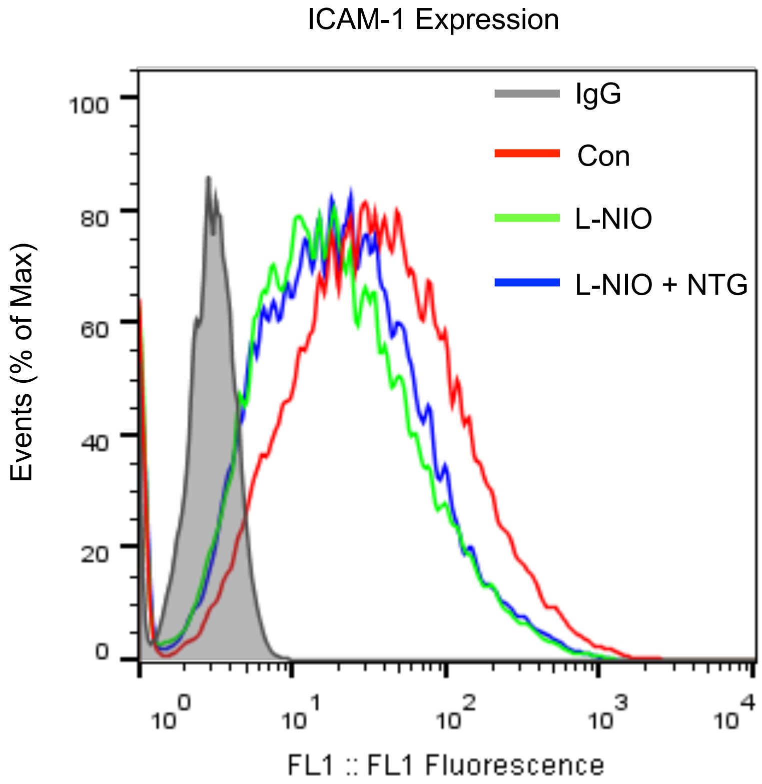

Supplementary Figure S1. ICAM-1 Expression is Not Altered by L-NIO or NTG Treatment. ECs were treated overnight with L-NIO (5 mM) ± NTG (5 μM) and surface expression of endothelial ICAM-1 was determined by flow cytometry. Quantitative analysis of fluorescence vs. size (electronic volume) histogram indicates that neither L-NIO nor NTG treatment alters ICAM-1 expression in ECs.

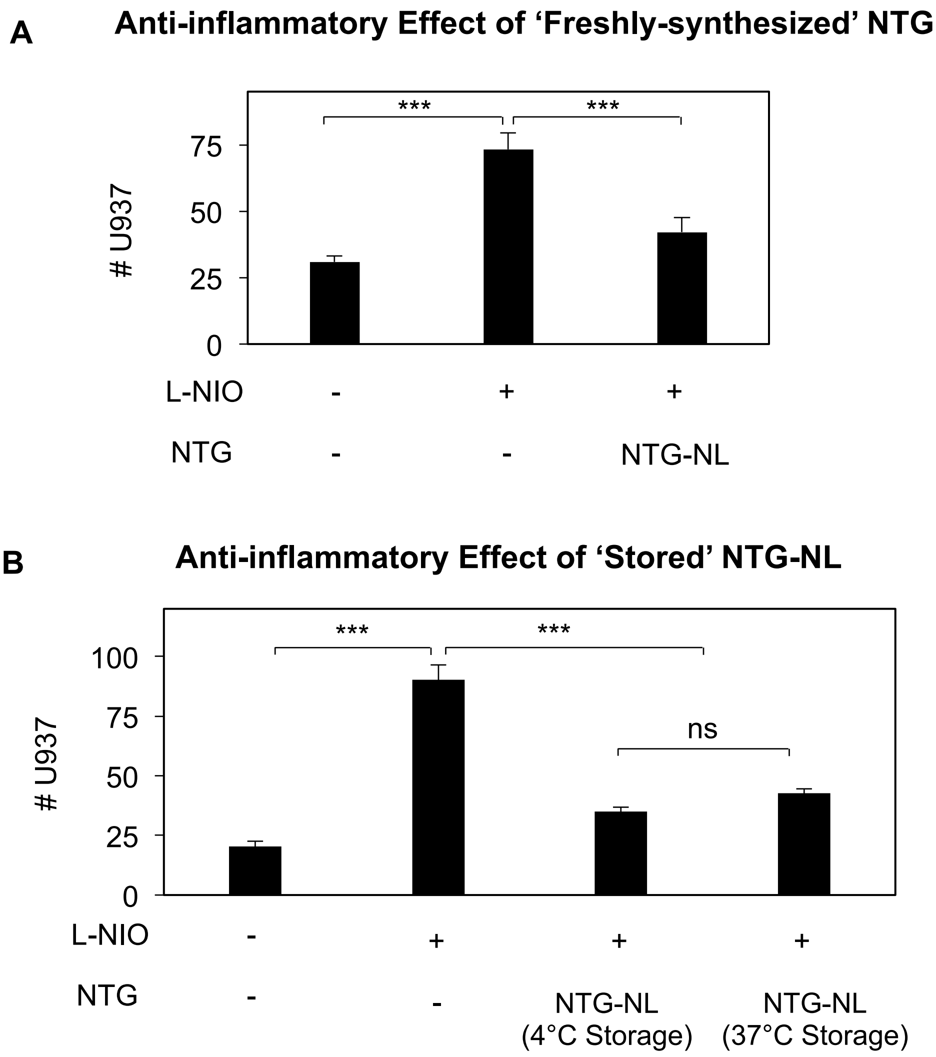

Supplementary Figure S2. Anti-inflammatory Effects of Freshly-prepared and Stored NTG-NL. EC monolayers were treated with L-NIO ± NTG-NL (100 µg/ml) that were either (**A**) freshly synthesized or **(B)** stored at 4°C or 37°C for 24 hr prior to use. Quantification of adherent U937 cells (per mm^2) on EC monolayers (n = 10 fields of view) reveals significant inhibition in U937 cell-EC adhesion following treatment with NTG-NLs that were stored for 24 hr, indicating stable retention of NTG within the lipid core of nanoliposomes. ***, p<0.001; ns, no significance.

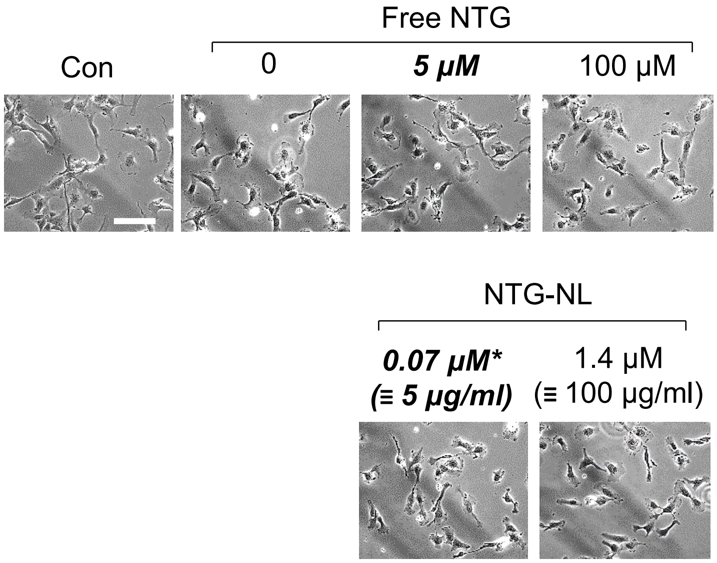

Supplementary Figure S3. EC Density And Spreading Are Similar Across Various Treatment Conditions. Phase images of ECs treated without or with L-NIO ± NTG or NTG-NL show that cell spreading is similar across all treatment conditions. Therefore, the difference in MitoSox® fluorescence intensity seen in Figure 7A reflects the actual difference in mitochondrial superoxide production. The effective therapeutic NTG dose is indicated ***in bold***. Scale bar = 100 µm

A

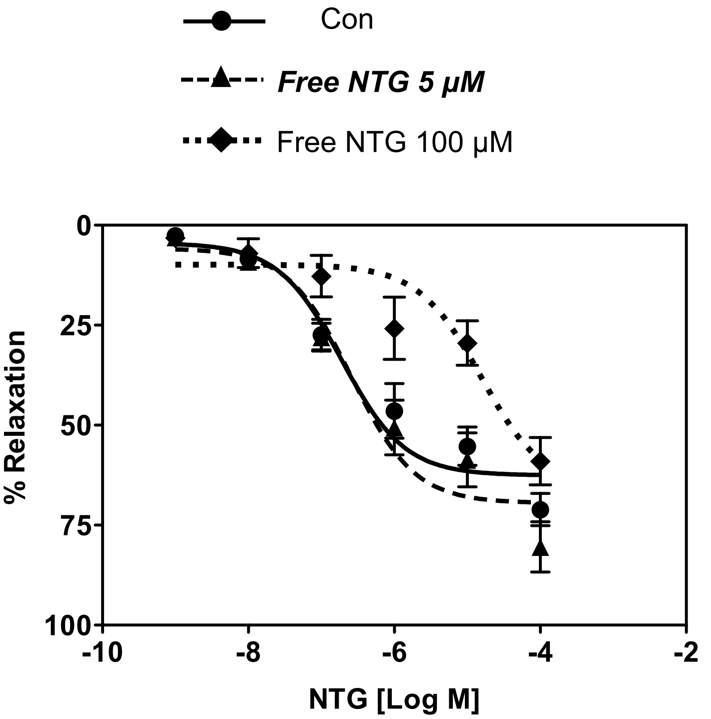

B

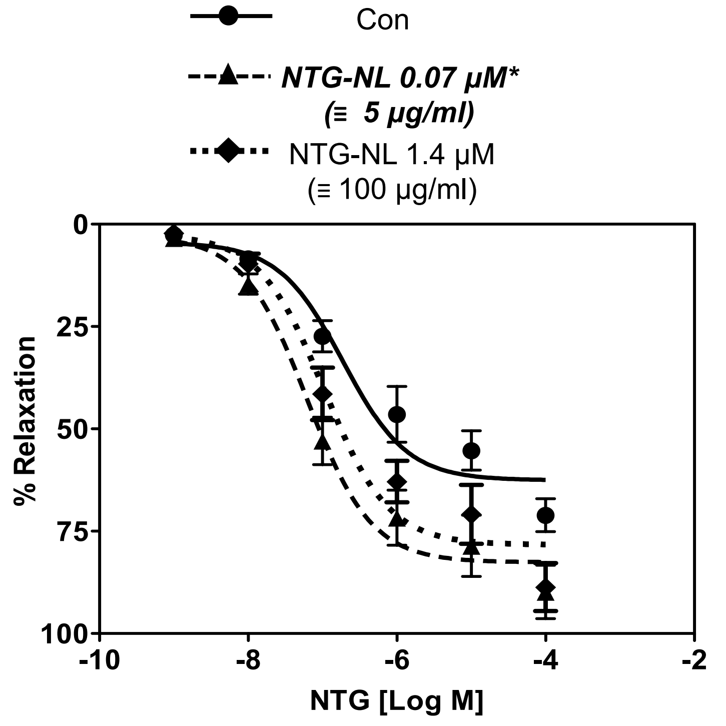

C

	-Log IC_{50} (Mean $IC_{50,}$ µM)		Maximal Relaxation (%)
Con	6.73 ± 0.14	(0.19)	63 ± 3
Free NTG 5 µM	**6.60 ± 0.18**	**(0.25)**	**69 ± 4***
Free NTG 100 µM	4.81 ± 0.24***	(15.5)	66 ± 9
NTG-NL 0.07 µM (5 µg/ml)	**7.21 ± 0.15**	**(0.07)**	**83 ± 3**
NTG-NL *1.4 µM* (100 µg/ml)	6.98 ± 0.15	(0.10)	78 ± 4

D

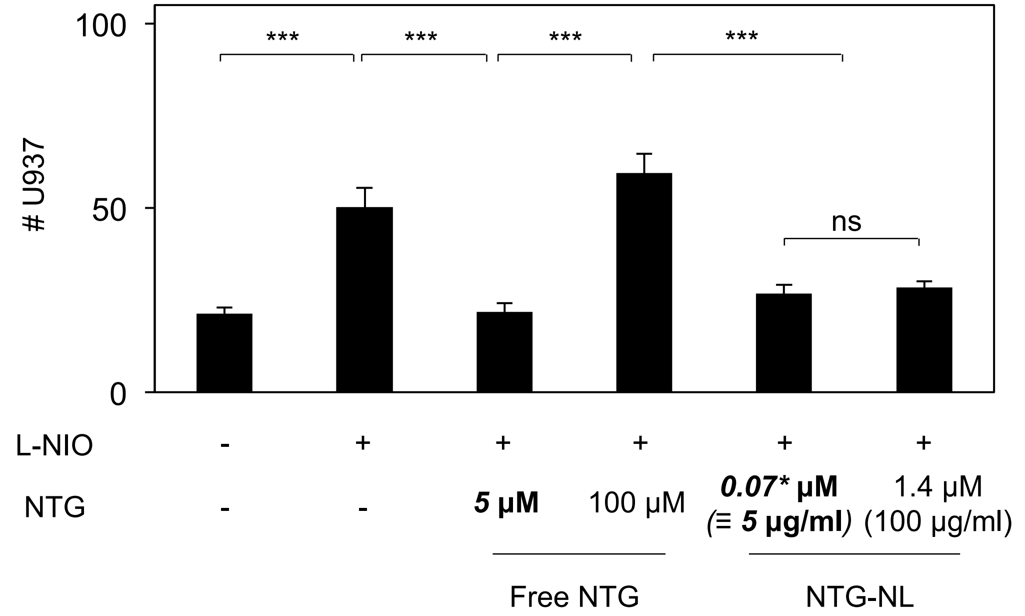

Supplementary Figure S4. Effects of Free NTG and NTG-NL Treatment on IC50 and Maximal Vasorelaxation of Isolated Pulmonary Sheep Arteries. (**A**) Pulmonary arterial rings pretreated with 100 μM free NTG exhibit impaired responsiveness to acute NTG treatment, as indicated by a significant rightward shift in NTG dose-relaxation curve (n ≥ eight arterial rings). (**B**) In contrast, arterial rings pretreated with a similar 20-fold higher NTG-NL dose exhibit normal relaxation response to acute NTG treatment. (**C**) Pulmonary sheep arteries pretreated with the effective (anti-inflammatory) free NTG dose of 5 μM exhibit normal NTG dose-relaxation profile similar to untreated controls. In contrast, arteries pretreated with a 20-fold higher free NTG dose (100 μM) demonstrate a significant increase in mean IC50 values. Remarkably, pretreatment with NTG-NL at both 5 and 100 μg/ml doses exhibit no evidence of NTG tolerance. IC50 are concentrations that produced 50% relaxation in response to NTG stimulation. The maximal relaxation response was, however, similar in the control and both free NTG-treated arteries while the NTG-NL-treated arteries exhibited marginally improved maximal relaxation response. The precise reason for this improvement in maximal relaxation by NTG-NL remains unclear. (**D**) Demonstration of intracellular NTG-NL uptake and bioavailability following 4h treatment. EC monolayers were treated with L-NIO ± NTG or NTG-NL for 4 hr prior to addition of fluorescently-labeled U937 monocytic cells. Quantification of adherent U937 cells (per mm^2) on EC monolayers (n = 10 fields of view) show that both NTG at 5 μM and NTG-NL at 5 μg/ml produce significant inhibition of U937 cell-EC adhesion within 4 hr of treatment. Further, while free NTG loses its therapeutic effect at a 20-fold higher dose of 100 μM, NTG-NL retains its immunosuppressive effects at a similar 20-fold greater dose (100 μg/mL). Thus, the anti-inflammatory effects of free NTG and NTG-NL observed after 4 hr of treatment are consistent with those seen following overnight treatment.
, p<0.01; ***, p<0.001; ns, no significance. Therapeutic dose is highlighted ***in bold*. Data are expressed as mean ± SEM.

ESI-MS Analysis of NTG Incorporation Efficiency within Nanoliposomes (NL)

Initial NTG Loading (% w/w)	Incorporated NTG (Peak Area)	NTG Incorporation Efficiency (%)
5	3039	15.6
10	14307	36.4
25	28653	23.4

Supplementary Table S1. ESI-MS Analysis of NTG Incorporation Efficiency within NLs. NTG incorporation within NLs increased with increasing loading (5, 10, and 25% wt. NTG/wt. NLs), although the maximum incorporation efficiency was observed at the intermediate NTG loading of 10% w/w (~37% incorporation efficiency). This trend is consistent with drug loading within nanoparticles, as previously reported.[1]

References:

1. Kilfoyle, B. E. *et al.* Development of paclitaxel-TyroSpheres for topical skin treatment. *J Control Release* **163**, 18-24, doi:Doi 10.1016/J.Jconrel.2012.06.021 (2012).

Nanoliposome (NL) Uptake by Cultured ECs

NL Conc. (µg/ml)	NL Uptake Net Fluor. Int. (A.U)	% NL Uptake
5	72	9.0
10	163	5.1
50	579	2.0
100	1580	0.8

Supplementary Table S2. NL Uptake by Cultured ECs. When added to cultured ECs, fluorescently-labeled nanoliposomes undergo dose-dependent uptake by ECs, with the net internalized amount increasing with increasing NL dose. However, this dose-dependent increase in net NL uptake is inversely proportional to *percent* uptake by ECs, which is the highest at 5 µg/ml dose and decreases progressively with increasing NL dose.
